# BECOMING DEMENTIA CAPABLE: AN INNOVATIVE EDUCATIONAL STRATEGY FOR EMS PROVIDERS

**DOI:** 10.1093/geroni/igae098.2169

**Published:** 2024-12-31

**Authors:** Kimberly Davis, Christy Jensen, Leland Waters

**Affiliations:** Virginia Commonwealth University, Richmond, Virginia, United States; Martha W. Goodson Center at Riverside Health System, Williamsburg, Virginia, United States; Virginia Commonwealth University, Richmond, Virginia, United States

## Abstract

There is an identified need to provide Emergency Medical Services (EMS) providers with foundational knowledge and skills in care of older adults living with dementia. EMS providers are uniquely positioned to recognize, intervene, and connect older adults and caregivers to community resources. Further, they can provide invaluable information and referrals to primary care providers (PCP), including partnering for long-term management. However, focus groups revealed they receive little to no formal training in best practices for care of older adults. This ongoing HRSA-funded project incorporates a two-pronged approach to training EMS providers: (1) training on the challenges of living with dementia from both the patient and caregiver perspectives including disease progression, management, communication skills, and community resources; and (2) training in advance care planning needs including introduction of the Physician Orders for Scope of Treatment, medical-ethical conversations in end of life, and patient advocacy. Content aligns with goals in the “Dementia State Plan 2020-2024: Building a Dementia Capable Virginia” which seeks to incorporate a public health approach to mitigate the impact of dementia. Training was provided in 7 regions of Virginia and has so far reached 275 EMS providers, first responders, and “hidden responders” (i.e. SW, behavioral health, elder law, etc.). Preliminary evaluation data reveals intention to change practice as a result of the educational strategy and will be shared at the symposium. As front-line workers, EMS providers are poised to help with early identification for older adults who would benefit from referral and early treatment, thereby reducing disease and disability.

